# Plasma LDL and HDL characteristics and carotenoid content are positively influenced by egg consumption in an elderly population^1^

**DOI:** 10.1186/1743-7075-3-6

**Published:** 2006-01-06

**Authors:** Christine M Greene, David Waters, Richard M Clark, John H Contois, Maria Luz Fernandez

**Affiliations:** 1 Department of Nutritional Sciences, University of Connecticut, Storrs, CT 06269, USA; 2 Liposcience, Inc, 2500 Sumner Blvd., Raleigh, NC 27616, USA

## Abstract

**Background:**

Approximately 1/3 of individuals have a high plasma response to dietary cholesterol (hyper-responders). Although increases in both LDL and HDL cholesterol have been observed, limited data exist regarding effects of egg consumption on lipoprotein subclasses and circulating carotenoids.

**Methods:**

29 postmenopausal women (50–68 y) and 13 men (60–80 y) were assigned to either 3 eggs (EGG, 640 mg cholesterol/d) or an equal volume of cholesterol-free egg substitute (SUB, 0 mg cholesterol/d) for 30 d. Following a 3 wk wash out, subjects crossed over to the alternate diet. Individuals with a response to dietary cholesterol > 2.2 mg/dL for each additional 100 mg of dietary cholesterol were classified as hyper-responders while hypo-responders were those with a response ≤ to 2.2 mg/dL. Nuclear Magnetic Resonance (NMR) spectroscopy determined LDL and HDL size & particle concentrations. Dietary records were used to evaluate carotenoid consumption.

**Results:**

Hyper-responders had higher concentrations of both LDL (LDL-C) and HDL (HDL-C) cholesterol after EGG. In contrast, the concentrations of plasma LDL-C and HDL-C did not differ between the EGG and SUB for the hypo-responders. After EGG, hyper-responders had larger (≥ 21.2 nm) less atherogenic LDL particle (P < 0.001) and larger HDL particle (> 8.8 nm) (P < 0.01), with no significant difference in the total number of LDL or HDL particles. Regardless of response classification, all individuals had an increase in plasma lutein (from 32.4 ± 15.2 to 46.4 ± 23.3 ng/L) and zeaxanthin (from 8.8 ± 4.8 to 10.7 ± 5.8 ng/L) during EGG, yet hyper-responders displayed higher concentrations of carotenoids when compared to hypo-responders

**Conclusion:**

These findings suggest that the increases in LDL-C and HDL-C due to increased egg consumption in hyper-responders are not related to an increased number of LDL or HDL particles but, to an increase in the less atherogenic lipoprotein subfractions. Also, increases in plasma carotenoids after EGG may provide a valuable dietary source for this population.

## Background

In the United States, people are living longer, healthier lives. Dietary requirements for the elderly must take into account the physiologic changes that occur with aging as these needs differ from those of younger adults. Nutritional support in the elderly is often a primary therapy and it has been shown that those that have an adequate intake of fatty acids and cholesterol as well as a greater intake of vitamins demonstrate higher intellectual function than those who have a deficient diet [1-3].

The habitual consumption of eggs has been shown to provide many nutritional benefits such as higher daily intakes of vitamins C, E and B12, in addition to folate [4]. Deficiencies in these vitamins have been associated with increased risk of cancer, cardiovascular disease and Alzheimer's disease [5-7]. Therefore, the addition of eggs to the diet could actually prevent disease development, particularly in this age demographic who are at elevated risks for the onset of chronic diseases. Additionally, current research has shown that eggs supply a significant amount of the carotenoids lutein and zeaxanthin, which have been linked to the prevention of age-related macular degeneration [8-11], the most common cause of blindness in those over the age of 60. On average, each egg yolk contains 292 μg of lutein and 213 μg of zeaxanthin, along with 0.7 mg vitamin E, 0.5 mcg vitamin B12, and 23.5 mcg folate, all in a highly bioavailable food matrix [12,13].

Because of the nutrient density of eggs, they are valuable contributors to overall nutritional balance, but they are also a source of dietary cholesterol. Currently, the relationship between plasma cholesterol and dietary cholesterol remains unclear. High serum cholesterol levels have been linked to increased coronary heart disease (CHD) [14,15] yet the risk of cardiovascular disease in men and women does not increase with increasing egg consumption [16-18] despite their high cholesterol content. Summarizing 166 cholesterol feeding studies conducted over 40 years on 3,500 subjects, a 100 mg/d increase in dietary cholesterol will raise total cholesterol 2.2 mg/dL or about 1% in normal responders [19]. This response relates to a 1.9 mg/dL change in low density lipoprotein (LDL) cholesterol and a 0.4 mg/dL increase in high density lipoprotein (HDL) cholesterol, utimately having little effect on the LDL/HDL cholesterol ratio, and minimal impact on CHD risk [20]. There is a large variation in response however, and hyper-responders are classified as those whose total cholesterol concentration will increase >2.2 mg/dL, and hypo-responders are those for whom total cholesterol increases < 2.0 mg/dL, for every 100 mg/d of dietary cholesterol consumed [21]. This heterogeneity in response confounds the relationship between dietary cholesterol and plasma cholesterol.

Plasma lipoproteins are comprised of a group of diverse particles with various physiochemical characteristics that define each subclass. It is the physiochemical characteristics that determine the degree of atherogenicity of the lipoprotein particle. In clinical practice, it is the cholesterol concentration carried in lipoproteins that captures the attention of the physician. This is the premise behind the use of the LDL and HDL cholesterol concentration ratio as an assessment of CHD risk. However, the evaluation of size deviation among lipoprotein subclasses has shown that normocholesterolemic individuals with a higher concentration of the small, dense LDL particle are at increased risk for coronary heart disease [22-24], despite similar LDL cholesterol concentrations. Similarly, when analyzing the cholesterol concentration of the HDL particle, the protective effects are often amplified when there is a higher concentration the larger subclass (HDL_2_) which is more antiatherogenic than the smaller HDL_3 _particle [25-28]. It has been documented that a dietary cholesterol challenge does not impact the LDL/HDL ratio in premenopausal women [29], or in an elderly population [30] comprised of both genders. In addition, traditional gel electrophoretic analysis of LDL particle size revealed that the larger LDL particle was predominant in hyper-responders during a dietary cholesterol intervention [31]. It can be surmised then that the cholesterol concentration that each subclass contains can vary by particle size, number, and density. Traditional lipoprotein cholesterol measurements, specifically the LDL/HDL ratio, fail to consider the variability in size distribution. This high degree of variability may therefore lead to inaccurate assessment of CHD risk.

The Adult Treatment Panel III recently acknowledged that the predominance of small dense LDL particles (sdLDL) is an emerging cardiovascular risk factor [32,33]. It has been reported that LDL size was the best predictor of coronary artery disease [34,35] and the increase in cardiovascular risk, attributable to lipid factors, was significantly modulated by variations in LDL particle size [32,36]. Utilizing nuclear magnetic resonance (NMR) spectroscopy, Blake, et al [37] found that median baseline values of LDL particle concentration were higher and LDL particle size was lower among women who subsequently experienced a coronary event. These evaluations suggest that the measurement of LDL cholesterol content alone is an inadequate marker of CHD risk in the general population. In a cohort of men from the Quebec Cardiovascular Study, the association between LDL particle size and the incidence of ischemic heart disease yielded similar results. In this study, multivariate analysis indicated that sdLDL particles predicted the rate of ischemic heart disease independent of LDL cholesterol content and other lipid risk factors [38]. This large scale, prospective evidence supports a hypothesis that LDL particle size is an important characteristic associated with CHD and that including LDL diameter analysis in CHD risk assessment may increase the accuracy of the evaluation over traditional lipid values.

Carotenoids and cholesterol share common pathways in absorption and transport in the plasma compartment as well as response differences. It is this commonality that led to this study, whose objectives are to evaluate the changes in lipoprotein size, cholesterol content and carotenoid concentration in an elderly population following egg consumption and relate those changes to their impact on CHD risk assessment.

## Methods

### Materials

Liquid whole eggs and cholesterol-free, fat-free egg substitute were purchased from Vistar (Windsor, CT). Enzymatic TC and triglyceride (TG) kits were obtained from Roche Diagnostics (Indianapolis, IN). Apolipoproteins (apo) B kits, EDTA, aprotinin, copper sulfate, sodium azide, phenylmethlysulfonyl fluoride (PMSF) were obtained from Sigma Chemical (St. Louis, MO).

### Subjects

Recruitment efforts were conducted through the use of local newspapers and brochure distribution within the surrounding University community. Men were required to be over the age of 60 while women were expected to be menopausal for at least one year. Recruitment of male participants was hindered due to the popular use of reductase inhibitor medication which excluded almost 60% of the male respondents. Ultimately, forty-two healthy volunteers (13 men and 29 women) were recruited with the exclusion criteria consisting of 1) allergy to eggs, 2) use of lipid-lowering medication, 3) history of heart disease, diabetes or kidney problems, 4) total cholesterol values higher than 240 mg/dL or total triglyceride levels over 300 mg/dL. The subjects were instructed to continue to consume their regular diet but to abstain from consuming eggs outside of those provided by the study. The study was conducted in accordance with the guidelines of the Institutional Review Board at the University of Connecticut.

### Experimental protocol

This study utilized a randomized cross-over design where subjects were initially assigned to an egg (EGG) or egg substitute (SUB) group for 30 days, followed by a three week washout period. The subjects were then crossed over to the alternate dietary intervention and continued for 30 days. Those assigned to the EGG group were expected to consume 3 eggs per day (approximately 640 mg dietary cholesterol). Conversely, the SUB group was given an equal volume of a cholesterol-free, fat-free product almost identical in color and consistency to the egg product (0 mg dietary cholesterol). Daily amounts were provided in individual containers and subjects were instructed to return any uneaten portion at the beginning of the following week. To ensure that diets during the intervention periods were equivalent and to calculate the amounts of dietary lutein and zeaxanthin, two seven-day dietary records were collected. Records included two weekend days and five non-consecutive weekdays. Nutrient intake was evaluated using Nutritional Data Systems software (NDS-R) Version 5.0, developed by the Nutrition Coordinating Center, University of Minnesota, Minneapolis, MN, Food and Nutrient Database 29. The variables of weight, waist and hip circumference, smoking and hormone replacement status were also measured at baseline and at the end of each treatment period to assess the influence these factors may have had on plasma lipids and lipoprotein characteristics.

Two fasting (12 h) blood draws were scheduled at the end of each intervention period, on non-consecutive days, 48 hours apart, for each subject. Whole blood was collected into tubes containing 0.10 mL/100 mL EDTA to determine plasma lipids. Plasma was separated by centrifugation at 1500 × g for 20 min at 4°C, and placed into vials containing PMSF (0.05 mL/100 mL), sodium azide (0.01 mL/100 mL) and aprotinin (0.01 mL/100 mL) and stored at 4°C until analysis. Plasma lipid analysis occurred within one week of collection.

### Classification of hyper- and hyporesponders

As previously described [39], a modest increase in total cholesterol of 0.05 – 0.06 mmol/L (2–2.2 mg/dL) may be considered a normal response to a 100 mg dietary cholesterol challenge. Therefore, because the subjects consumed an additional 640 mg/d of dietary cholesterol, those that had an increase in total cholesterol (TC) of ≥ 0.41 mmol/L (16 mg/dL) were labeled hyper-responders and those whose fluctuations in TC were ≤ 0.36 mmol/L (14 mg/dL), were identified as hypo-responders [29,40,41].

### Plasma lipids

Since 1989 our laboratory has participated in the Center for Disease Control/National Heart, Lung and Blood Institute (CDC-NHLBI) lipid standardization program for quality control and standardization of plasma TC, TG and HDL-C assays. The coefficients of variance assessed by the standardization program during this study were 0.76–1.2% for TC, 1.64 – 2.47% for TG and 1.71 – 2.27% for HDL-C. Plasma lipids were determined by averaging three values obtained from the separate blood draws.

Analysis included enzymatic TC, as described by Allain et al [42] and triglyceride (TG) calculations [43] with kits from Roche Diagnostics that adjust for free glycerol. High density lipoprotein cholesterol content (HDL-C) was evaluated after the precipitation of apolipoprotein-B containing particles with a magnesium chloride and dextran sulfate solution [44]. Low density lipoprotein cholesterol content (LDL-C) was determined using the Friedewald equation [45].

### Plasma lutein and Zeaxanthin determinations

Plasma (200 μl) was prepared for HPLC analysis by combining each sample with an internal standard of 50 μl ethyl-β-apo-8'-carotenoate (Fluka, Ronkonkoma, NY) and 200 μl ethanol containing butylated hydroxytoluene, similar to the method previously described [46]. Briefly, the sample was extracted three separate times using a hexane carrier containing butylated hydroxytoluene while centrifugation facilitated the phase separation. The solvent was removed with a stream of nitrogen and the resulting hexane layers were reconstituted with 100 μl of 2-propanol and placed into HPLC injection vials. A Waters HPLC system equipped with a Varian column (100 × 4.6 mm Microsorb-MN 100-3 C-18), and preceded by an Upchurch C-18 guard column (Upchurch Scientific, Oak Harbor, WA) was used to analyze the carotenoid content of the plasma. The isocratic mobile phase contained 80% acetonitrile, 15% dioxane, 2.5% methanol, 2.5% 2-propanol, 0.01% triethylamine, and 0.01% ammonium acetate. The internal standard and carotenoid content of the plasma was detected at 450 nm. All solvents were HPLC grade and were filtered and degassed before use. Standard curves were compiled from HPLC purified lutein and zeaxanthin.

### Lipoprotein size determinations

Based on the natural proton NMR profiles of lipoprotein particles, a spectroscopic alternative to separation-based methodology was utilized to analyze lipoprotein subclasses [47-49]. This analysis took place at Liposcience, Inc. and will be briefly described here. An intermediate field (400 MHz) NMR analyzer (Bruker BioSpin Corp, Billerica, MA) with an automated flow-injection process was used to measure the lipoprotein subclasses in plasma samples. The deviations to be seen between lipoprotein subclasses are the result of the diameter of the phospholipid shell of the particle and are not influenced by apolipoproteins contained within the shell. The amplitude of each particle's signal is a measure of the concentration of the lipoprotein subclass. NMR simultaneously quantifies > 30 lipoprotein subclasses that were empirically grouped into 10 smaller subclasses based on particle diameter. The ten subclasses analyzed in this study were large VLDL (>60 nm), medium VLDL (35–60 nm), small VLDL (27 – 35 nm), large LDL (21.2 – 23 nm), medium LDL (19.8 – 21.2 nm), small LDL (18 – 19.8 nm), very small LDL (16.8 – 18 nm), large HDL (8.8 – 13 nm), medium HDL (8.2 – 8.8 nm) and small HDL (7.3 – 8.2 nm).

### Statistical analysis

Repeated measures analysis of variance (ANOVA) was used to analyze the effects of egg consumption on plasma lipids, HDL, VLDL, and LDL particle characteristics and lutein and zeaxanthin concentrations. Each individual's response to diet (egg or substitute) was considered as the repeated measure with the response classification used as the between subject factor. P < 0.05 was considered significant. Data are presented as means ± SDs for the number of subjects in each group. Statistical analysis was conducted using SPSS v. 12 for Windows (SPSS, Chicago).

## Results

### Study population characteristics

There was no difference between hyper- and hypo-responders with respect to baseline values of TC, LDL-C, and TG or body mass index (data not shown). Body mass index values did not differ between response groups during the EGG or SUB periods (Hyper-responders; 27.3 + 6.3 EGG, 26.9 + 5.9 SUB: Hypo-responders; 27.2 + 4.7 EGG, 27.2 + 4.9 SUB)

### Plasma lipid concentrations

Plasma concentrations for the hyper-responders showed significant diet effects on plasma lipids (Table 1). Total cholesterol, LDL-C and HDL-C were higher after the EGG period compared to the SUB period for hyper-responders (P < 0.0001) (Table 1). In contrast, total cholesterol, LDL-C or HDL-C were not different between the EGG and SUB period for hypo-responders. There was no statistical significance to changes in triglycerides as a result of diet or response effects during this study (Table 1).

**Table 1:** Plasma concentrations of total cholesterol LDL-C, HDL-C and TG of male and female hyper- and hypo-responders during the EGG and placebo (SUB) periods^1^

	Total Cholesterol	LDL-C	HDL-C	TG
	mg/dL			
***Hyper-responders (n = 15)***				
EGG	210.6 ± 43.3^a^	130.8 ± 45.4^a^	60.3 ± 13.6^a^	97.5 ± 62.8
SUB	175.6 ± 41.4^b^	103.4 ± 28.0^b^	54.6 ± 10.5^b^	95.3 ± 75.2
***Hypo-responders (n = 27)***				
EGG	178.6 ± 27.6^b^	101.9 ± 39.4^b^	55.3 ± 14.9^b^	99.9 ± 42.2
SUB	181.5 ± 25.7^b^	106.2 ± 24.6^b^	54.2 ± 14.5^b^	106.0 ± 42.5
Diet Effect	P < 0.0001	P < 0.0001	P < 0.0001	NS^2^
Response Effect	P < 0.0001	P < 0.0001	P < 0.01	NS

^1 ^Values are shown as the mean ± SD. Values in the same column with different superscript are significantly different as determined by repeated measures ANOVA

^2 ^NS = non-significant

### Lipoprotein particle size

**Figure 1:**
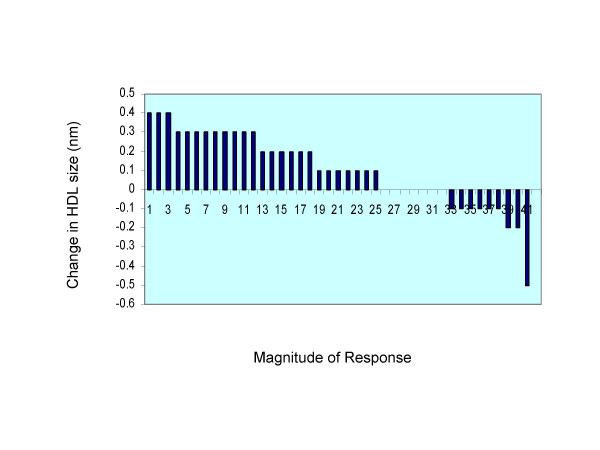
Changes in HDL particle size as a result in egg consumption. Each bar represents one subject.

Comparison of the average VLDL, LDL and HDL particle size in hyper- and hypo-responders revealed statistical significance only for HDL showing a larger HDL particle seen during the EGG treatment period for both hyper and hypo-responders (Table 2). VLDL and LDL particle size was not affected by diet or response classification. The changes in HDL size as a result of egg consumption are indicated in Fig. 1. As seen in the figure the majority of the subjects had an increase in HDL particle size following EGG period, independent of response classification.

**Table 2:** VLDL, LDL and HDL Particle Sizes of male and female hyper- and hypo-responders during the EGG and placebo (SUB) periods^1^.

	VLDL	LDL	HDL
	(nm)		
***Hyper-responders (n = 15)***			
EGG	46.05 ± 8.78	21.77 ± 0.95	9.49 ± 0.55
SUB	48.60 ± 8.55	21.48 ± 0.95	9.35 ± 0.49
***Hypo-responders (n = 27)***			
EGG	47.30 ± 7.39	21.37 ± 0.81	9.19 ± 0.46
SUB	46.94 ± 7.35	21.41 ± 0.80	9.10 ± 0.49
Diet Effect	NS^2^	NS	P < 0.01
Response Effect	NS	NS	NS

^1 ^Values are presented as mean ± SD.

^2 ^NS = non-significant

**Figure 2:**
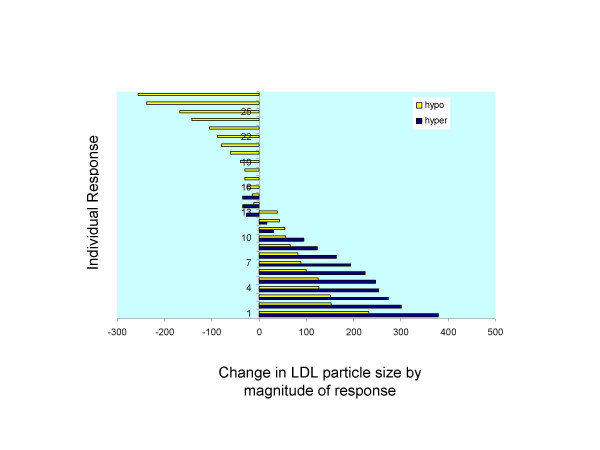
Changes in concentration of the large LDL subclass due to egg consumption and classification response. Each bar represents one hyper (blue bars) or hypo (yellow bars).

Individual subclass analysis of the concentration of LDL particles showed a significant increase in the larger LDL particle during the EGG treatment period for hyper-responders (Table 3). Particle concentration of the large LDL subclass was 674.7 ± 258.4 μmol/L during the EGG period and 521.1 ± 267.3 μmol/L during the SUB period for hyper-responders for a diet effect of P < 0.0001. Comparing response classifications, it was noted that hyper-responders had an increased concentration of the larger LDL particle (674.7 ± 258.4 μmol/L for EGG period) than the concentrations in hypo-responders (543.3 ± 252.2 μmol/L for EGG period) demonstrating a significant response effect. The changes in the large LDL subclass due to egg intake are illustrated in Fig. 2. The diet effect and the response effect in increasing LDL size are clearly depicted for all subjects.

**Table 3:** Concentration of total LDL and LDL sub-fractions of male and female hyper- and hypo-responders during the EGG and placebo (SUB) periods^1^.

	Total LDL	Large LDL	Medium LDL	Small LDL	Very Small LDL
	(nmol/L)				
***Hyper-responders (n = 15)***					
EGG	1241 ± 633.	674.7 ± 258.4^a^	101.5 ± 127.3	525.9 ± 696.0	491.7 ± 355.7
SUB	1186 ± 529	521.1 ± 267.3^b^	130.7 ± 134.0	629.3 ± 633.9	517.4 ± 321.2
***Hypo-responders (n = 27)***					
EGG	1217 ± 320	543.3 ± 252.2^b^	128.7 ± 86.6	637.3 ± 426.0	424.3 ± 570.1
SUB	1209 ± 356	548.1 ± 229.4^b^	124.4 ± 82.0	624.6 ± 415.9	498.3 ± 501.2
Diet Effect	NS^2^	P < 0.0001	NS	NS	NS
Response Effect	NS	P < 0.0001	NS	NS	NS

^1 ^Values are shown as the mean ± SD. Values in the same column with different superscript are significantly different as determined by repeated measures ANOVA

^2 ^NS = non-significant

The concentration of HDL particles also showed a significant response effect as hyper-responders had an increase in the larger HDL particle concentration when compared to hypo-responders (Table 4). There were no differences seen for hypo-responders in HDL subclass particle sizes when comparing dietary interventions.

**Table 4:** Concentration of total HDL and HDL sub-fractions of male and female hyper- and hypo-responders during the EGG and placebo (SUB) periods^1^.

	Total HDL	Large HDL	Medium HDL	Small HDL
	μmol/L			
***Hyper-responders (n = 15)***				
EGG	34.3 ± 7.1	10.44 ± 5.07^a^	3.06 ± 4.54	20.83 ± 3.74
SUB	33.1 ± 5.2	8.47 ± 3.58^b^	3.86 ± 3.99	20.76 ± 3.75
***Hypo-responders (n = 27)***				
EGG	35.6 ± 6.6	8.70 ± 3.90^b^	2.40 ± 2.45	24.58 ± 5.05
SUB	36.6 ± 7.1	8.90 ± 4.89^b^	1.94 ± 2.05	25.79 ± 6.11
Diet Effect	NS^2^	NS	NS	NS
Response Effect	NS	P < 0.025	NS	NS

^1 ^Values are shown as the mean ± SD. Values in the same column with different superscript are significantly different as determined by repeated measures ANOVA

^2^NS = non-significant

### Carotenoid analysis

Analysis of the dietary carotenoids, lutein and zeaxanthin, yielded a significant diet effect as there was an increased consumption of both carotenoids during the EGG treatment period. Dietary amounts were 4655 ± 1989 μg and 3429 ± 2003 μg for hyper-responders during the EGG and SUB treatment periods, respectively, while hypo-responders consumed 4857 ± 3048 μg during the EGG period and 2755 ± 1608 μg during the SUB treatment (Table 5)

**Table 5:** Dietary and plasma concentrations of lutein + zeaxanthin of male and female hypo- and hyper-responders during the EGG and placebo (SUB) periods

	Dietary Lutein + Zeaxanthin	Plasma Lutein	Plasma Zeaxanthin
(nm)	(μg/dL)	(μg/ml)	
***Hyper-responders (n = 15)***			
EGG	4655 ± 1989^a^	55.3 ± 26.3^a^	14.3 ± 6.7^a^
SUB	3429 ± 2003^b^	35.3 ± 16.6^c^	10.5 ± 6.4^b^
***Hypo-responders (n = 27)***			
EGG	4857 ± 3048^a^	41.5 ± 20.3^b^	8.7 ± 4.0^b^
SUB	2755 ± 1608^b^	30.7 ± 14.4^c^	7.8 ± 3.5^c^
Diet Effect	P < 0.0001	P < 0.0001	P < 0.01
Response Effect	NS	P < 0.05	P < 0.05

^1 ^Values are shown as the mean ± SD. Values in the same column with different superscript are significantly different as determined by repeated measures ANOVA

^2 ^NS = non-significant

**Figure 3:**
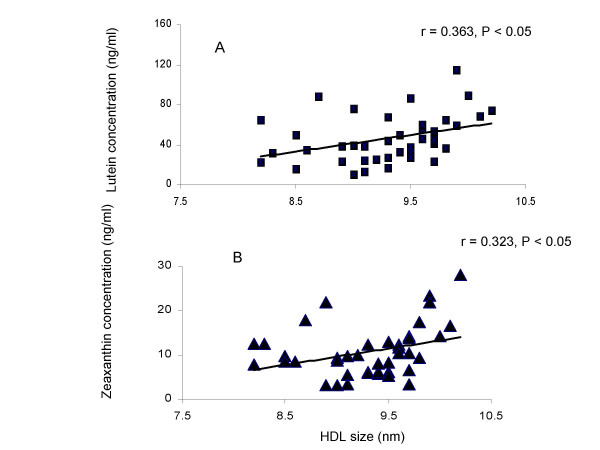
Correlation between large HDL concentrations and plasma lutein for all subjects during the egg period.

Plasma evaluation of these carotenoids also revealed a diet effect in lutein and zeaxanthin concentrations as all subjects increased plasma levels during the EGG period (Table 5). Although absolute plasma concentrations for zeaxanthin were lower than lutein, the response was similar with an increased plasma concentration seen during the EGG treatment period with significant elevation noted in the hyper-responders for both carotenoids. A significant correlation between HDL size and plasma lutein (r = 0.361, P < 0.05) and zeaxanthin (r = 0.321, P < 0.05) was observed for all subjects during the egg period (Fig. 3) indicating that the higher concentration of plasma carotenoids were related to the larger HDL following egg intake.

## Discussion

While there is little doubt that there exists a continuum of increasing risk for developing CHD with increasing plasma cholesterol concentrations, there is uncertainty in the mechanisms that link hypercholesterolemia and atherosclerosis. For any given LDL cholesterol concentration there is great variability in the clinical expression of the disease. Patients with heterozygous familial hypercholesterolemia exhibit a wide array of ages at which they develop CHD and even patients with homozygous familial hypercholesterolemia may differ by as much as 30 years in the age at which CHD is clinically expressed [50]. This striking variability implies a complex and multifaceted mechanism between LDL cholesterol and the development of atherosclerosis. Clearly, the consideration of plasma cholesterol alone or ratios that consider only the cholesterol content of lipoproteins are inadequate indicators of CHD risk. Because the cholesterol content of the LDL subclass varies with particle size and number, and is derived by calculation not direct measurements, the inherent use of this value in the estimation of CHD risk is fraught with errors. The expression of multiple distinct LDL subclasses which differ in pathogenic roles, offers the opportunity to classify the particle based on its phenotype. Previous studies have suggested that sdLDL are part of a cluster of dyslipidemias, termed the atherogenic lipoprotein phenotype [51]. Prospective work has also reported that the sdLDL phenotype is a significant predictor of CVD [34,36,52]. It stands to reason then that an evaluation of particle size and number should be included in the assessment of CHD risk. In this study, with an elevation of plasma cholesterol as a result of the dietary intervention in the hyper-responders, the analysis of not only changes in lipoprotein cholesterol content but also size and number of lipoprotein subclasses were useful to elucidate the mechanisms behind the changes seen in TC levels.

While a significant diet effect was seen in TC, LDL and HDL cholesterol for hyper-responders during the EGG period, it is valuable to note that there was a concomitant increase in the large LDL particle concentration in this sub-population. Elevated levels of large LDL are classified as Pattern A and are considered less atherogenic. The smaller LDL phenotype, Pattern B, is considered more atherogenic. This small, dense LDL phenotype is recognized as a strong coronary risk factor independent of traditional lipoprotein cholesterol ratios [53]. If the small, dense LDL particle is considered pro-atherogenic, as documented elsewhere, then changes in the large LDL particle may be considered less atherogenic in this population. This implies that the relationship between changes in plasma cholesterol levels and increases in CHD risk is incomplete and that the modification of the lipoprotein particle outside of its cholesterol concentration is critical to the understanding of the mechanism behind elevated TC. By measuring the particle size and number, through the efficient NMR technology, we can now visualize the metabolic processes that might occur during a dietary cholesterol challenge and speculate on the mechanisms involved. Because the LDL cholesterol content increased significantly, it was important to analyze the subclass of LDL particle that carried the changes in cholesterol concentration. NMR data indicated that only the larger LDL particle increased during the EGG period with hyper-responders demonstrating a significantly larger increase than hypo-responders. A larger, more buoyant LDL particle is hypothesized to have a reduced susceptibility to both oxidation and endothelial penetration. This suggests that a less atherogenic LDL particle is responsible for the increase in LDL cholesterol seen in this study.

Similarly, HDL cholesterol content was elevated during the EGG period for hyper-responders. HDL is considered the 'good' cholesterol and elevations in this lipoprotein are generally considered antiatherogenic. This too, relates to the functionality of the particle as the HDL particle serves to remove excess cholesterol from the body and return it to the liver for excretion. Analyzing the particle size of the major classes of lipoproteins, this study shows that there was a significant increase in HDL lipoprotein size as a result of the EGG treatment, where VLDL and LDL showed no significant changes. The analysis of HDL subclasses demonstrated a significant increase in the large HDL particle for hyper-responders. This indicates a cardioprotective effect in this population as the larger HDL particle has been correlated with decreased CHD risk [54]. Hyper-responders are classically defined as individuals who react to dietary cholesterol with a large increase in plasma cholesterol. The response shown here indicates that a portion of the increase in TC is associated with an increase in the larger, antiatherogenic HDL particle and would therefore offset the effects of increases in LDL cholesterol. We surmise from this that egg consumption will increase the cardioprotective HDL subclasses as a means of managing the dietary cholesterol and of balancing cholesterol homeostasis in these elderly subjects.

The lipid content of the yolk provides a highly bioavailable matrix for the absorption of lutein and zeaxanthin [12]. Because the SUB diet did not contain additional lutein and zeaxanthin, these carotenoids where found to significantly increase in the plasma as a result of the EGG treatment. Interestingly, hyper-responders had a larger increase in carotenoid plasma response, mimicking their elevated cholesterol response. This suggests a common pathway, given that both cholesterol and these carotenoids where elevated as a result of the administration of this diet. The chemical characteristics of lutein and zeaxanthin would not allow incorporation into the hydrophobic core of lipoproteins but instead permit the free association with the lipoprotein phospholipid shell. It is known that these carotenoids are carried primarily in the HDL subclass [55-57] and we have shown that the larger HDL is increasing as a result of this intervention. We suspect that some of the increased carotenoid intake has found its way into the plasma compartment coincident with the changes to the HDL particle by means of the reverse cholesterol transport system. Due to the location of lutein and zeaxanthin on the surface of lipoproteins, it is possible that these carotenoids could exchange during particle remodeling, while HDL interacts with other lipoproteins in the plasma compartment. There is tissue specificity with respect to lutein and zeaxanthin concentration with the macular pigment having the greatest concentration[46]. Because plasma levels of these carotenoids increase following changes in diet and supplementation, it is proposed that the antioxidant nature of these carotenoids could serve to decrease the oxidative stress potential found in the plasma compartment and thereby reduces oxidative damage. This is the hypothesis behind the use of lutein and zeaxanthin in the prevention of macular degeneration and might also represent the mechanism by which they could offer cardioprotective effects.

## Conclusion

Taken together these findings suggest that (1) LDL particle numbers do not significantly increase despite an increase in LDL-C with egg consumption; and (2) lipoprotein size is positively altered by the consumption of three eggs per day in elderly subjects. The data shown here displays a significant increase in the larger LDL and HDL particles for hyper-responders, which suggests an antiatherogenic profile. In addition, the increase in plasma lutein and zeaxanthin following egg consumption may provide a valuable source of functional carotenoids in the diet for the population. Within the nutritional community there is a developing appreciation that health derives, not from avoidance of particular foods, but from an overall pattern of diet, and it has been suggested that dietary recommendations should shift from one of avoidance to promotion. This study suggests that egg consumption may be permitted, and perhaps promoted, in a healthy elderly population to supplement carotenoid intake with minimal reservations concerning coronary heart disease risk.

## List of Abbreviations used

CHD: coronary heart disease; HDL-C: HDL cholesterol; HPLC: high performance liquid chromatography; LCAT: lecithin cholesterol acyltransferase; LDL-C: LDL cholesterol; NMR: Nuclear Magnetic Resonance; sdLDL: small dense LDL particle; TC: total cholesterol; TG: triglycerides; VLDL: very low density lipoprotein.

## Competing interests

Authors received funding from the American Egg Board/Egg Nutrition Center to carry out these studies.

## Authors' contributions

CMG was involved in the acquisition, analysis and interpretation of data in addition to drafting the manuscript, DMW made significant contributions to the acquisition and analysis of the data, RMC was involved in interpretation of data and critical analysis of intellectual content of the manuscript, JHC contributed to the design, acquisition and interpretation of the data, and MLF was instrumental in the study's inception, design and approval while providing critical analysis of data interpretation and manuscript review.
